# Supplementary material for: Learning curve and surgical outcome of robotic assisted colorectal surgery with ERAS program
Source: Sci Rep. 2022 Nov 29;12:20566. doi: 10.1038/s41598-022-24665-w (PMC9709162; doi:10.1038/s41598-022-24665-w)
Supplement: Supplementary file 1 — Supplementary Information 1. [file 41598_2022_24665_MOESM1_ESM.zip › LSC Raw data-Ñ[▒K/144/O_ñw╝╨░O▒Kñσ.pdf]

正式摘要(電子簽章：000000229803898)

請選擇...reporter

臺中榮民總醫院

Operation Note

[redacted]

- 手術日期：2021/11/15
- 手術醫師：VS 蔣鋒帆，CR 林陳彥，R 林志安，R 蔡濬樟
- Anesthesia : GE
- Position : Lithotomy
- Aseptic agents : Betadine Aqua , Zephnan

Diagnosis

- Pre-operative diagnosis : Malignant tumor of sigmoid colon with obstruction
- Post-operative diagnosis : Locally advanced S-colon tumor with liver metastasis and peritoneal seeding s/p laparoscopic anterior resection

Surgical Indication

- For reliving the obstruction symptoms

Operation

- 1. Laparoscopic anterior resection
  2. Intraoperative colonoscopy : (-)
  3. High ligation (+) : At IMA root
  4. EndoGIA (60-4.1) X1 were applied for the initial resection. Then another staple of EndoGIA (60-4.1) was applied over the upper rectal stump under the concern of the poor blood supply condition. The anastomosis was completed via firing EEA (29mm).

  # In short, totally 2 staples of EndoGIA was applied.
  5. Proximal cut end: 5 cm, distal cut end: >5 cm
  6. No drainage tube was placed into the abdominal cavity
  7. The ADHESION BARRIER FILM was then placed below the central extended wound.
  8. The colon was not taken down from the splenic flexure site

Finding

- 1. A long segment, ulcerative tumor at distal sigmoid colon, at least 4cm X 5cm in size. Much residule stool was noted, which was compatible with her obstructive condition.
  2. The tumor was locally advanced and invaded to the nearby (left) gonadal vessel.
  3. Seeding lesions were presented over the nearby S-colon and on the small bowel mesentery.
  4. Multiple enlarged LNs were all over the IMA area.
  5. Some dirty (Brownish) ascites were presented at pelvic area.
  6. Bilateral doughnuts : intact ; Air leak test: Done and no air leak was found

Procedure

- 1. General endotracheal anesthesia was induced and then the patient was placed in modified Trendelenburg lithotomy position. Preoperative antibiotics were given. A Foley catheter and a nasogastric tube were placed. The abdomen was prepped and draped in standard sterile fashion.
  2. A small vertical incision was made right next to the umbilicus and a 12 mm trocar was inserted into the peritoneal cavity by open method. Peumoperitoneum was established with CO_2 inflation. Laparoscopy was inserted and then another 12 mm trocar was inserted over RLQ. Extra three 5 mm trochars were inserted over RUQ, LUQ and LLQ of abdomen. The operative findings were

- mentioned as above.
  3. The small bowel was inspected and retracted to the right by grasper. The sigmoid colon was then held by grasper and the peritoneum overlying the mesentery was then scored with electrocautery.
  4. The inferior mesentery artery was identified, ligated below the left colic artery with endoclips, and transected, so was inferior mesentery vein. Multiple LNs were identified. Dissection was done above the Gerota's fascia and toward the peritoneal attachments.
  5. The arcade vessels to the sigmoid colon were also divided. The left gonadal vessel was en-bloc resected.
  6. The left broad ligament was also partially invaded by the tumor. En-bloc resectio was performed. Several endoclips were applied for bleeding control.
  7. After the adhesion was freed, distal descending colon and upper rectum were mobilized.
  8. EndoGIA (60-4.1) was applied for resection.
  9. However, under the concern of the poor blood supply status over the upper rectal stump, another EndoGIA (60-4.1) was applied to perform the resection.
  10. Incision was further extended from the umbilical 12 mm trochar wound. The proximal colon was then taken out for carefully inspection, and to make sure it has an excellent blood supply. The mesenteric fat was gently cleared from the terminal 2-3 mm of the bowel ends.
  11. The field was isolated with moist pad and the bowel opened and sized. It was determined that a 29 mm circular stapler could be accommodated. A pursestring suture of 2-0 prolene was placed, the anvil was inserted, and then the pursestring suture was tied.
  12. The anal canal was irrigated with distil water and then the circular stapler was introduced through the anus and pierced through the mid-portion of the stapler line.
  13. The proximal bowel with anvil in place was engaged with the circular stapler and the stapler was then fired. Two complete donuts were obtained and the distal one was submitted to pathology for margin confirmation.
  14. The anastomosis was tested for leaks with irrigation solution and air introduced through the rectum and was found to be patent and intact.
  15. The possible bleeder was then checked thoroughly.
  16. The ADHESION BARRIER FILM was then placed below the central extended wound.
  17. The fascia of midline wound was closed with interrupted sutures of Num. 1 Vicryl. The fascia of the 12 mm trochar was also with 1-0 Vicryl.
  18. After massive irrigation, the skin wound was sutured with interrupted method via 3-O Vicryl, then covered with Surgicel and Medipore.
  19. The patient tolerated the procedure well and was brought to the POR in stable condition.
- Estimated blood loss: minimum
- 器械數量: 正確
- 紗布數量: 正確

Image

- PAINT
  - abdomen

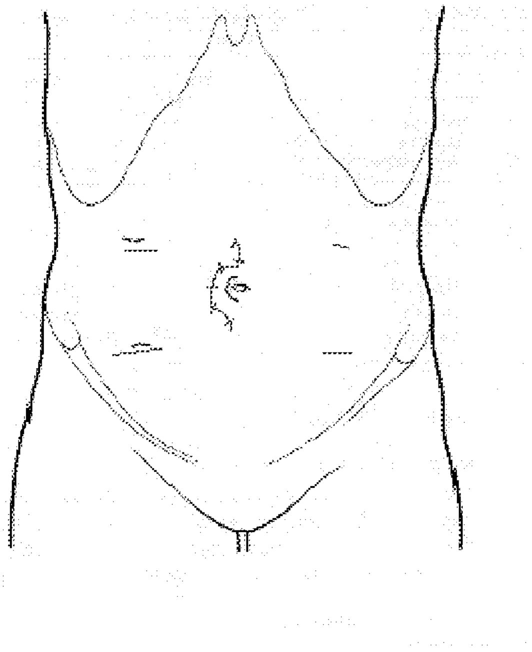

- colon & rectum

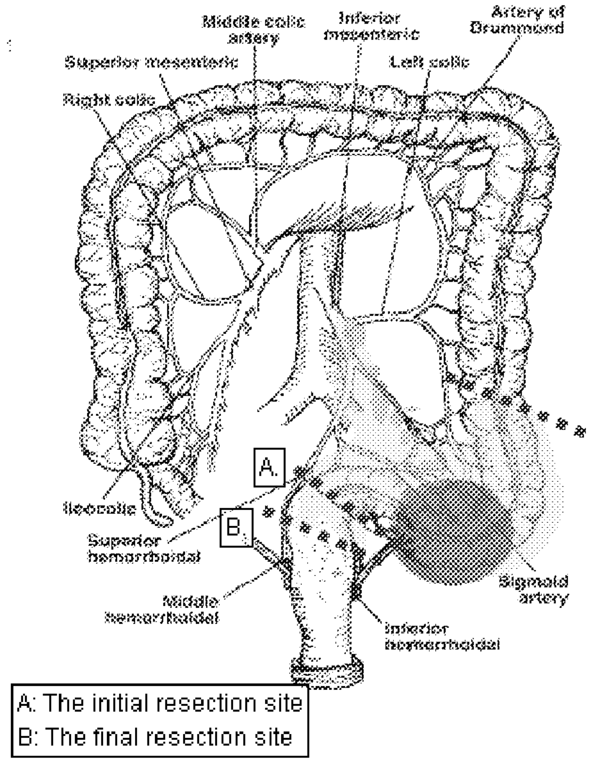

- The resected S-colon. Seeding nodules could be easily seen.

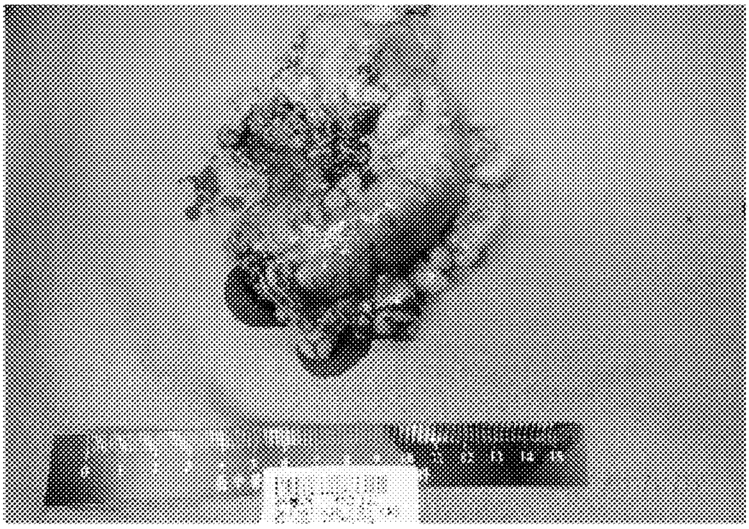

Notice : The further resected, a small segement of distal S-colon was not presented in the picture

Enlarged lymphnodes were all over the IMA area.

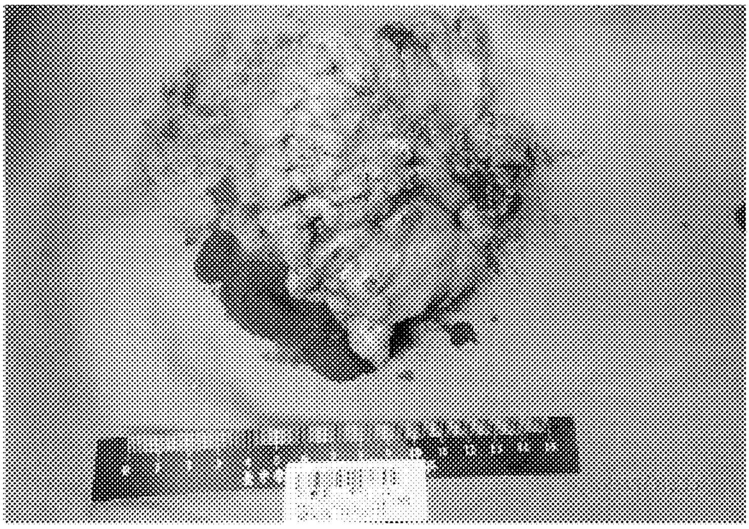

The ulcerative tumor.

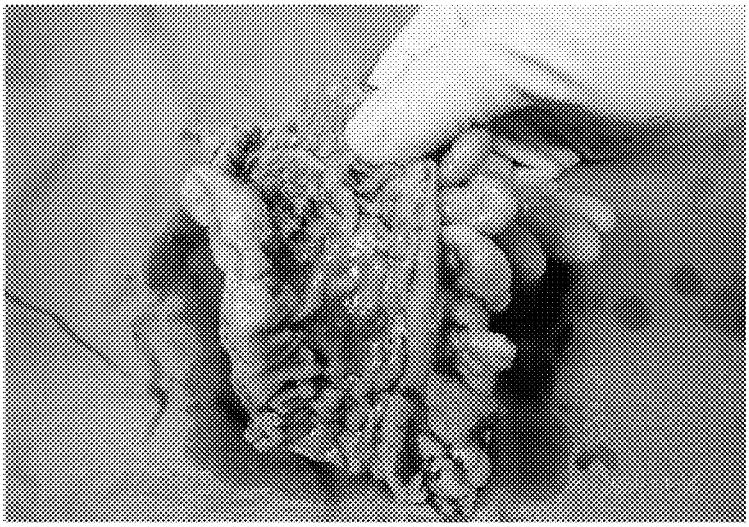

主任 2062F蔣鋒帆 / 醫師 2301H林陳彥

正式摘要**(**電子簽章：**000000229803898)**

請選擇...reporter

doctorRank:B
